# Erratum for Randell et al., “Opportunistic dried blood spot sampling validates and optimizes a pediatric population pharmacokinetic model of metronidazole”

**DOI:** 10.1128/aac.00972-25

**Published:** 2025-07-25

**Authors:** Rachel L. Randell, Stephen J. Balevic, Rachel G. Greenberg, Michael Cohen-Wolkowiez, Elizabeth J. Thompson, Saranya Venkatachalam, Michael J. Smith, Catherine Bendel, Joseph M. Bliss, Hala Chaaban, Rakesh Chhabra, Christiane E. L. Dammann, L. Corbin Downey, Chi Hornik, Naveed Hussain, Matthew M. Laughon, Adrian Lavery, Fernando Moya, Matthew Saxonhouse, Gregory M. Sokol, Andrea Trembath, Joern-Hendrik Weitkamp, Christoph P. Hornik, Best Pharmaceuticals for Children Act – Pediatric Trials Network Steering Committee

## ERRATUM

Volume 68, no. 4, e01533-23, 2024, https://doi.org/10.1128/aac.01533-23. Page 6, Table 1, row 7: “7” should read “15.7.” This typographical error for the Hill parameter estimate did not impact any modeling code or analysis, and the results, conclusions, and clinical relevance of the study are unchanged.

